# Supplementary material for: From sequence to enzyme mechanism using multi-label machine learning
Source: BMC Bioinformatics. 2014 May 19;15:150. doi: 10.1186/1471-2105-15-150 (PMC4229970; doi:10.1186/1471-2105-15-150)
Supplement: Additional file 2 — Java code of ml2db. Additional file ml2db_code.tar.gz contains the Java source code to run the multi-label machine learning experiments and save the results to database. The code’s Javadoc is included. [file 1471-2105-15-150-S2.zip › additional file 2/ml2db/ml2db/doc/mulan/transformations/multiclass/package-summary.html]

mulan.transformations.multiclass


JavaScript is disabled on your browser.


- Overview
- Package
- Class
- Use
- Tree
- Deprecated
- Index
- Help

- Prev Package
- Next Package

- Frames
- No Frames

- All Classes

# Package mulan.transformations.multiclass

- Interface Summary

  | Interface | Description |
  | --- | --- |
  |  |  |
  | --- | --- |
  | MultiClassTransformation | The interface for single-label multi-class transformations. |
- Class Summary

  | Class | Description |
  | --- | --- |
  |  |  |
  | --- | --- |
  | Copy | Class that implement the Copy transformation method |
  | CopyWeight | Class that implement the Copy-Weight transformation method |
  | Ignore | Class that implement the Ignore transformation method |
  | MultiClassTransformationBase | The base class for multi-class transformation methods. |
  | SelectBasedOnFrequency | Class that implement the Select-Max and Select-Min transformation methods. |
  | SelectRandom | Class that implement the Select-Random transformation method |
- Enum Summary

  | Enum | Description |
  | --- | --- |
  |  |  |
  | --- | --- |
  | SelectionType |  |

- Overview
- Package
- Class
- Use
- Tree
- Deprecated
- Index
- Help

- Prev Package
- Next Package

- Frames
- No Frames

- All Classes
